# PLA2R1 and HLA-DQA1 SNP in patients with primary membranous nephropathy

**DOI:** 10.1371/journal.pone.0328234

**Published:** 2025-08-22

**Authors:** Junyi Zhou, Zhijian Zhang, Kezhi Zhou, Leting Zhou, Jing Xue, Bin Liu, Xiran Zhang, Ting Cai, Biao Huang, Yi Zhang, Zhigang Hu, Liang Wang, Xiaobin Liu

**Affiliations:** 1 Department of Nephrology, The Affiliated Wuxi People’s Hospital of Nanjing Medical University, Wuxi People’s Hospital, Wuxi Medical Center, Nanjing Medical University, Wuxi, P.R. China; 2 College of Life Sciences and Medicine, Zhejiang Sci-Tech University, Hangzhou, P.R. China; 3 NHC Key Laboratory of Nuclear Medicine, Jiangsu Key Laboratory of Molecular Nuclear Medicine, Jiangsu Institute of Nuclear Medicine, Wuxi, P.R. China; 4 Medical Laboratory, The Affiliated Wuxi Children’s Hospital of Nanjing Medical University, Wuxi, China; The University of North Carolina at Charlotte, UNITED STATES OF AMERICA

## Abstract

**Background:**

Primary membranous nephropathy is a widely recognized autoimmune disease associated with podocyte antigens; the most important autoantigen is PLA2R1. *PLA2R1* and *HLA-DQA1* play important roles in the production of pathogenic antibodies. The purpose of this study was to observe the relationship between gene polymorphisms and primary membranous nephropathy and explore the clinical functional clues of *PLA2R1* and *HLA-DQA1* genes affecting treatment responsiveness.

**Method:**

The study enrolled 89 patients with primary membranous nephropathy and 91 healthy people as a control. Single-nucleotide polymorphism loci (seven on *PLA2R1* and two on *HLA-DQA1*) were identified using the PCR-Sanger technique. The patients were followed up until the 12th month, and relevant clinical data were collected. The relationship between these single-nucleotide polymorphism loci and primary membranous nephropathy remission was analyzed.

**Result:**

Genotypic and allelic frequency distributions for six single-nucleotide polymorphisms within *PLA2R1* (rs4664308, rs3792189, rs3792192, rs1870102, rs17831251, and rs35771982) and one in *HLA-DQA1* (rs2187668) were associated with morbidity of primary membranous nephropathy. Single-nucleotide polymorphisms rs1870102, rs17831251, and rs2187668 were statistically significant in the genetic model analysis. The odds ratio for primary membranous nephropathy in patients carrying rs2187668 GG and rs1870102 AA was 52.875. We found that *PLA2R1* single-nucleotide polymorphism rs36771982 was related to proteinuria remission at the 12^th^ month, and found in further analysis that *PLA2R1* single-nucleotide polymorphisms rs3792189, rs3792192, rs17831251, and rs35771982 were related to treatment response in the RTX group.

**Conclusion:**

In this study, we found several *PLA2R1* and *HLA-DQA1* single-nucleotide polymorphism loci associated with primary membranous nephropathy morbidity and that some *PLA2R1* single-nucleotide polymorphism loci were related to the treatment response of patients with primary membranous nephropathy.

## Introduction

Membranous nephropathy (MN) is a common nephrotic syndrome (NS) in adults. According to etiology, MN can be divided into secondary membranous nephropathy (SMN) and primary membranous nephropathy (PMN), which accounts for about 80% of all MN. At present, PMN is believed to be related to the autoimmune response associated with podocyte antigens, the most common of which is the M-type phospholipase A2 receptor (PLA2R), concurrently, emerging scholarship demonstrates that non-invasive serological profiling can discriminate PLA2R-associated membranous nephropathy from both non-PLA2R-related MN and other nephrotic syndromes with high fidelity [1,2]. The pathological process of anti-PLA2R antibody production remains unclear, but the major histocompatibility complex (MHC) plays an important role in antigen presentation.

Various studies have explored the relationship between *PLA2R1* and *HLA-DQA1* polymorphisms and PMN. As early as 1979, the HLA gene was discovered to be related to MN [3], and a 4.5 Kb DQA fragment length polymorphism was proposed as a momentous disease susceptibility factor for PMN in 1989 [4]. In 2010, Liu discovered a relationship between *PLA2R1* single-nucleotide polymorphism (SNP) and MN, in which the G allele and GG genotype of rs35771982 increased the risk of MN [5]. In 2011, a genome-wide association study (GWAS) of MN in Caucasian populations confirmed that the SNP loci of *PLA2R1* and *HLA-DQA1* were related to MN in individuals of white ancestry, in which the top SNP loci were rs2187668 and rs4664308 [6]. In 2020, a GWAS involving East Asian and European cohorts not only reported that the SNP of *PLA2R1* and *HLA-DQA1* were associated with MN, but also identified two novel genes associated with MN, *NFKB1* and *IRF4*. Racial differences in HLA alleles between East Asia and Europe patients were also mentioned in this study, including DRB1*1501 in East Asian patients and DQA1*0501 in European patients [7].

The 2021 KDIGO guidelines established a requirement to combine various biomarkers for the risk prediction of MN and suggested establishing a gene clinical risk score for recurrence after transplantation [8]. In addition to being associated with the PMN pathogenesis, which has been demonstrated previously, *PLA2R1* and *HLA-DQA1* SNP loci may also have clinical functional clues. Related studies may contribute to our understanding of the pathogenesis and disease characteristics of PMN.

This study aimed to determine the relationship between PMN and *PLA2R1* and *HLA-DQA1* SNP polymorphisms, and to explore the functional role of *PLA2R1* and *HLA-DQA1* genes in response to treatment in patients with PMN.

## Materials and methods

### Study population

This study was a retrospective study and included 89 patients diagnosed with PLA2R-associated PMN by renal biopsy between 2021–2022 at Wuxi People’s Hospital,who are all Han Chinese..The exclusion criteria were: 1. secondary membranous nephropathy; 2. malignant tumor, systemic lupus erythematosus, rheumatoid arthritis, hepatitis B, or other systemic immune diseases; 3. older than 80 years or not Han Chinese; and 4. digestive, respiratory, circulatory, blood, and other serious diseases. Patients with PMN were treated as follows: 44 (49.4%) received CTX therapy, 34 (38.2%) received RTX therapy, nine (10.1%) received CNIs therapy, and two (2.2%) did not receive immunosuppressive therapy. All patients received maximal renal support therapy based on ACEI/ARB applications. Meanwhile, 91 healthy people who underwent physical examination at Wuxi People’s Hospital were included in the control group.

### Sample collection and testing

Blood samples were collected before renal biopsy and at 12 months follow-up, and were centrifuged at 3000 rpm for 4 min. The supernatant was taken and stored at −80°C waiting for detection. In addition, whole-blood samples were collected from PMN patients and healthy people for sequencing. Genomic DNA was extracted from the peripheral blood samples. Seven SNP loci on *PLA2R1* and two SNP loci on *HLA-DQA1* were identified using PCR-Sanger sequencing.(DNA extraction from whole blood samples was performed by centrifugation column method, and DNA quality was verified by agarose gel electrophoresis) PLA2R-IgG was quantitatively detected using EUROIMMUN ELISA.

### Criteria for proteinuria remission

Complete remission was defined as proteinuria less than 0.3 g per day and a stable glomerular filtration rate. Partial remission was defined as proteinuria less than 3.5 g per day and reduced by at least 50%, serum albumin > 35 g/L, and stable renal function.

Non-remission was defined as the failure to reach the standard of complete or partial remission.

### Statistical analysis

Clinical data were analyzed using IBM SPSS Statistics 27. The population genetics Hardy–Weinberg balance test, linkage disequilibrium (LD) degree, and haplotype analysis were performed using the Shesis online software. Odds ratios (OR), standard errors, 95% confidence intervals (95%CI), and correlation analyses were performed using MedCalc software. The calculated p-value was based on Sheskin (2004) (Sheskin DJ (2004) Handbook of Parametric and Nonparametric Statistical Procedures. 3rd ed. Boca Raton: Chapman & Hall/CRC). When P < 0.05, the differences were considered statistically significant.

### Ethical approval

The experimental protocol was developed based on the ethical guidelines of the Declaration of Helsinki and approved by the Human Ethics Committee (Wuxi People’s Hospital Ethics Committee). This study was approved by the ethics committee of our hospital (approval no. kyl2016001).

## Results

### Clinical characters at baseline and 12th month

Of the 89 patients, 76 achieved partial or complete clinical remission at 12 months follow-up (Table 1). There were no significant differences in gender and age between the remission and non-remission groups(P > 0.05). At the same time, there was no statistically significant difference in serum albumin level, proteinuria, serum creatinine and PLA2R-IgG at baseline, serum creatinine, or eGFR at the 12th month between the remission and non-remission groups (P > 0.05). Significant differences were observed in serum albumin, proteinuria, PLA2R-IgG at the 12th month and baseline eGFR between the two groups at the 12th month.

**Table 1:** Clinical data of Baseline and 12-month follow-up in PMN patients.

	Remission(n = 76)	Non-Remission(n = 13)	*P*
Male(%)^●^	45(59.21%)	8(61.54%)	0.874
Age, year^△^	50.89 ± 13.45271	55 ± 12.14496	0.306
**Baseline**			
Serum albumin, g/L^△^	22.85 ± 4.33965	22.5692 ± 5.82114	0.839
Proteinuria, g/d^*^	4.77(3.89, 5.86)	5.09(4.095, 6.75)	0.332
Serum creatinine, umol/L^*^	73.1(61.125, 88.625)	87.3(60.35, 130.2)	0.248
eGFR, mL/min/1.73 m^2^^*^	95.5(83.775, 108.925)	84.4(49.8, 98.95)	0.037
PLA2R-IgG, RU/mL^a^^,^^*^	60.52(14.5250, 171.6725)	62.85(15.2225, 288.1675)	0.739
**12th Month**			
Serum albumin, g/L^△^	37.89 ± 5.34619	27.15 ± 7.05149	<0.01
Proteinuria, g/d^*^	0.435(0.17, 1.515)	3.77(2.675, 5.045)	<0.01
Serum creatinine, umol/L^*^	72.35(62.55, 84.675)	79.3(51.85, 156.8)	0.396
eGFR, mL/min/ 1.73 m^2^^*^	95.85(84.325, 106.35)	79.7(39.5, 109.75)	0.335
PLA2R-IgG, RU/mL^a^^,^^*^	1.77(1.3650, 3.1300)	30.71(6.2500, 65.1900)	0.02

● Analyzed using the chi-square test, and described as number(frequency).

△ Analyzed using student’s t-test and described as mean and standard deviation.

* Analyzed using the Mann-Whitney U test and described as interquartile range (IQR).

^a^ PLA2R-IgG was detected by EUROIMMUN ELISA.

For further analysis, we divided the patients into different subgroups according to the treatment plan. Here we compared the clinical data of CTX group and RTX group at baseline and 12 months of follow-up(Table 2).There was no statistical difference in gender and age distribution between the two groups, and there was no statistical difference in serum albumin, 24-hour proteinuria, serum creatinine, eGFR, pla2r IgG at baseline and the 12th month.At the same time, there was no significant difference in remission between the two groups.

**Table 2:** Clinical data of baseline and 12-month follow-up in RTX and CTX groups.

	RTX(n = 34)	CTX(n = 44)	P
Gender^●^	19(55.9%)	24(54.5%)	0.91
Age^*^	54.00(41.00,59.00)	54.00(41.00,63.00)	0.84
Baseline			
Serum albumin, g/L^*^	22.70(19.30,25.40)	23.20(19.40,24.70)	0.75
Proteinuria, g/d^*^	4.32(3.65,5.79)	4.83(4.10,6.09)	0.66
Serum creatinine, umol/L^△^	81.40 ± 42.96	89.1 ± 74.68	0.6
eGFR, mL/min/1.73 m2^*^	95.10(75.5,105.00)	94.80(83.50,109.70)	0.74
PLA2R-IgG, RU/mLa^△^^,^^a^	129.96 ± 190.47	152.35 ± 261.75	0.7
12-months			
Serum albumin, g/L^*^	37.35(33.23,42.00)	36.65(31.30,40.52)	0.37
Proteinuria, g/d^△^	1.36 ± 1.41	1.29 ± 1.51	0.84
Serum creatinine, umol/L^*^	70.05(54.13,83.50)	70.15(65.28,81.85)	0.53
eGFR, mL/min/1.73 m2^*^	99.45(85.23,108.30)	96.60(72.43,103.45)	0.98
PLA2R-IgG, RU/mLa^△^^,^^a^	7.24 ± 12.37	38.84 ± 149.73	0.25
Remission^●^	29(85.3%)	38(86.4%)	0.57

● Analyzed using the chi-square test, and described as number(frequency).

△ Analyzed using student’s t-test and described as mean and standard deviation.

* Analyzed using the Mann-Whitney U test and described as interquartile range (IQR).

^a^ PLA2R-IgG was detected by EUROIMMUN ELISA.

### Genotype and gene frequency in PMN and healthy control groups

The genotype and gene frequency distributions of *PLA2R1* SNP rs4664308, rs3792189, rs3792192, rs1870102, rs17831251, and rs35771982, and *HLA-DQA1* SNP rs2187668 were significantly different (P < 0.05) in PMN patients and the healthy population (Table 3).

**Table 3:** Genotype and gene frequency in PMN and healthy control groups.

	Genotype	PMN	Control	Total	OR(95%CI)^*^	*P*	Allele	PMN	Control	Total	MAF	OR(95%CI)	*P*
**PLA2R**													
rs4664308	AA	87	80	167	5.9813(1.2862-27.8151)	0.0226	A	176	161	337	0.05693	11.4783(2.6495-49.7269)	0.0011
	AG	2	1	3			G	2	21	23			
	GG	0	10	10									
rs3792189	CC	45	21	66	3.4091(1.7963-6.4698)	0.0002	C	118	84	202	0.43889	2.2944(1.4985-3.5132)	0.0001
	CA	28	42	70			A	60	98	158			
	AA	16	28	44									
rs3792192	GG	80	66	146	3.367(1.4701-7.7115)	0.0041	G	166	136	302	1.6111	4.6789(2.3833-9.1855)	<0.0001
	GA	6	4	10			A	12	46	58			
	AA	3	21	24									
rs1870102	AA	81	49	130	8.6786(3.7648-20.0057)	<0.0001	A	162	129	291	0.19167	4.1599(2.2715-7.6181)	<0.0001
	AG	0	31	31			G	16	53	69			
	GG	8	11	19									
rs17831251	CC	69	81	150	0.0208(0.0012-0.3502)	0.0072	C	138	162	300	0.16667	0.4259(0.2378-0.7629)	0.0041
	CT	0	0	0			T	40	20	60			
	TT	20	10	30									
rs3828323	CC	79	73	152	1.9479(0.8443-4.494)	0.118	C	162	156	318	0.11667	1.6875(0.8718-3.2664)	0.1205
	CT	4	10	14			T	16	26	42			
	TT	6	8	14									
rs35771982	GG	81	70	151	3.0375(1.2664-7.2858)	0.0128	G	167	149	316	0.12222	3.3624(1.6412-6.8888)	0.0009
	GC	5	9	14			C	11	33	44			
	CC	3	12	15									
**HLA-DQA1**													
rs2187668	GG	67	83	150	0.2935(0.1229-0.7013)	0.0058	G	145	171	136	0.10891	0.305(0.1481-0.6281)	0.0013
	GA	11	5	16			A	33	11	44			
	AA	11	3	14									
rs28383345	GG	65	57	122	1.6155(0.8586-3.0397)	0.1369	G	150	146	296	0.17778	1.3209(0.7668-2.2756)	0.3158
	GA	20	32	52			A	28	36	64			
	AA	4	2	6									

* OR value and 95% confidence interval for wild-type alleles.

Compared with healthy controls, the G gene frequency of rs4664308(A > G), A gene frequency of rs3792189(C > A), A gene frequency of rs3792192 (G > A), G gene frequency of rs1870102(A > G), and C gene frequency of rs35771982(G > C) were lower in the PMN group (*P* < 0.05). In contrast, the T gene frequency of rs1781251(C > T) and A gene frequency of rs2177668(G > A) were higher than in the control group (P < 0.05) (Table 3).

There were no statistically significant differences in the genotypes or gene frequencies of the PLA2R1 SNP rs3828323 and HLA-DQA1 SNP rs28383345 loci between the two groups (P > 0.05).

### Genetic risk model analysis

*PLA2R1* SNP rs1870102 and rs17831251 and *HLA-DQA1* SNP rs2187668 were used for the genetic model analysis. The risk model analysis of *PLA2R1* SNP rs1870102 showed different results by model: in the explicit model, patients carrying the AA genotype had a higher risk of PMN than those carrying the AG or GG genotype (OR=8.786, 95%CI 3.7648–20.0057, P < 0.0001); in the additive model, patients carrying the A allele had a higher risk of PMN than patients who carried the G allele (OR=93.1983, P = 0.0061), and there was no significant difference in the implicit model (P = 0.5001). The risk model of *PLA2R1* SNP rs17831251 showed that patients carrying the TT genotype had a higher risk than patients who carrying the CC genotype in both the explicit and implicit models (OR=0.4259, 95%CI 0.1868–0.9712, P = 0.424) as a result of the absence of the CT genotype in the PMN group, and there was no significant difference in the additive model (P = 0.9912). The risk model analysis of *HLA-DQA1* SNP rs2187668 showed that patients who carried the GG or GA genotype had a lower risk of PMN than those carrying the AA genotype in the implicit model (OR=0.2417, 95%CI 0.0651–0.9882, P = 0.034), although there was no significant difference in the explicit or additive model (P = 0.0912 in explicit model and P = 0.1146 in additive model) (Table 4).

**Table 4:** Gene risk model analysis of SNP rs1870102, rs17831251 and rs2187668.

		OR^*^	95%CI	*P*
rs1870102				
Explicit model	AA vs. (AG + GG)	8.6786	3.7648-20.0057	<0.0001
Implicit model	(AA + AG) vs. GG	1.3722	0.5321-3.6422	0.5001
Additive model	A vs. G	93.1983	5.5956-1552.2835	0.0061
rs17831251				
Explicit model	CC vs. (CT + TT)	0.4259	0.1868-0.9712	0.0424
Implicit model	(CC + CT) vs. TT	0.4259	0.1868-0.9712	0.0424
Additive model	C vs. T	0.9781	0.0192-49.8346	0.9912
rs2187668				
Explicit model	GG vs. (GA + AA)	1.9974	0.8949-4.4584	0.0912
Implicit model	(GG + GA) vs. AA	0.2417	0.0651-0.8982	0.034
Additive model	G vs. A	0.4123	0.1371-1.2392	0.1146

* OR denotes odds ratio.

### Interaction between *PLA2R1* and *HLA-DQA1* genes

Next, *PLA2R1* SNP rs1870102 and *HLA-DQA1* SNP rs2187668 were used in the gene interaction analysis, as shown in Table 5. Patients who carried the GG of *HLA-DQA1* rs2187668 and AA of *PLA2R1* SNP rs1870102 had an odds ratio 52.875 times (8.46/0.16) higher than those who did not carry the risk alleles.

**Table 5:** Interaction between *PLA2R1* SNP rs1870102 and *HLA-DQA1* SNP rs2187668^*^.

		rs1870102(*PLA2R1*)		
rs2187668(*HLA-DQA1*)		AA	AG	GG
PMN/Total	GG	62/89	0/89	5/89
Odds ratio (95% CI)		8.46(4.2894 to 16.6857)	1(0.0196 to 50.9546)	0.16(0.039-0.79)
PMN/Total	GA	9/89	0/89	2/89
Odds ratio (95% CI)		0.0266(0.0111 to 0.0633)	1(0.0196 to 50.9546)	0.3103(0.0609 to 1.5819)
PMN/Total	AA	10/89	0/89	1/89
Odds ratio (95% CI)		0.0321(0.0139 to 0.0741)	1(0.0196 to 50.9546)	0.1331(0.0160 to 1.1055)

* Odds ratio for PMN based on SNP and genotype combinations.

### Correlations of SNP with proteinuria remission at 12th months

**Fig 1:**
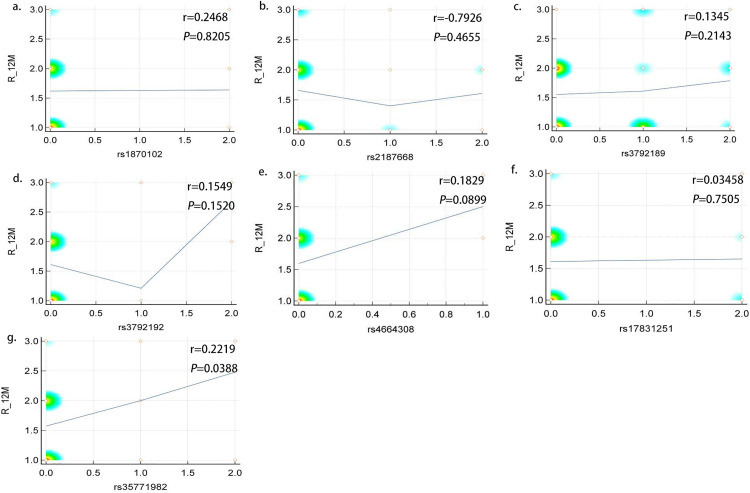
The relationship between SNPs and treatment response in patients with PMN.

We conducted a correlation analysis between the seven SNP loci associated with PMN and 12th month proteinuria remission in 87 patients who received various immunosuppressive therapies and found that *PLA2R1* SNP rs35771982 was significantly associated with proteinuria remission (r = 0.2219, P = 0.0388), whereas the other six SNP loci were not (Fig 1).

**Fig 2:**
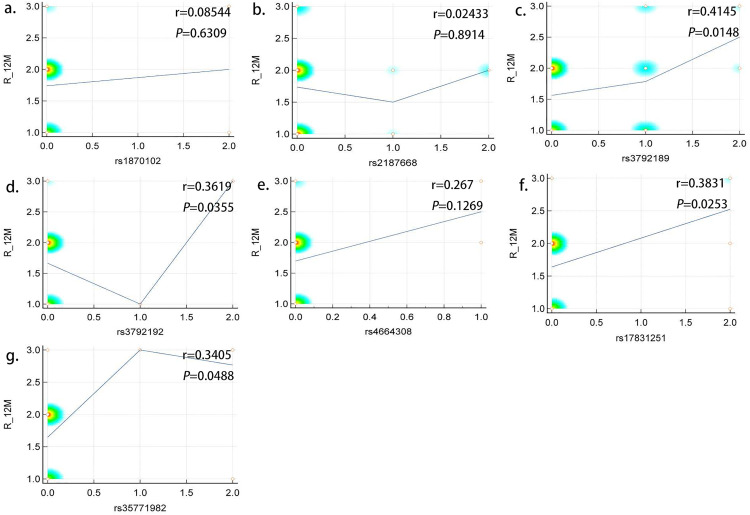
The relationship between SNPs and treatment response in RTX group. Spearman correlation analysis was performed using the MedCalc software.

Subsequently, to further analyze the correlations between SNP loci and proteinuria remission, we divided all patients with PMN into various groups according to immunosuppressive therapy regimens, including the CTX (n = 44) and RTX groups (n = 34) (Fig 2). The results demonstrated that within the RTX group, four SNP loci on PLA2R1 were associated with treatment response at the 12th month, including rs3792189 (r = 0.4145, P = 0.0148), rs3792192 (r = 0.3619, P = 0.0355), rs17831251 (r = 0.3831, P = 0.0253), and rs35771982 (r = 0.3405, P = 0.0488). However, none of the seven SNP loci was associated with treatment responsiveness in the CTX group (P > 0.05).

## Discussion

It is well known that the etiology of PMN is related to the production of pathogenic antibodies against podocyte antigens, which results from the joint effect of genetic and environmental factors [9,10]. The aim of this study was to demonstrate the relationship between the *PLA2R1* and *HLA-DQA1* genes and PMN and to determine the clinical value of SNP in the treatment of primary membranous nephropathy.

This study involved nine SNP loci in *PLA2R1* and *HLA-DQA1* in a PMN cohort. Compared with healthy people, PMN patients had statistically different genotypes and allele frequencies in seven out of nine SNP loci (P < 0.05), illustrating that these SNP loci, including *PLA2R1* SNP loci rs4664308, rs3792189, rs3792192, rs1870102, rs17831251, and rs35771982, and *HLA-DQA1* SNP locus rs2187668, are related to PMN. These results indicate that *PLA2R1* and *HLA-DQA1* are associated with PMN pathogenesis. Our results support the theory that *PLA2R1* SNP affects the conformation of PLA2R, increasing the susceptibility of its antigen to abnormal exposure to the immune system and PLA2R epitopes recognized and presented by MHC class II molecules encoded by the HLA gene, in which *HLA-DQA1* plays an important role in stimulating the production of pathogenic antibodies.

The relationship between PMN and SNP in different populations has been previously discussed. Stanescu et al.found that *PLA2R1* and *HLA-DQA1* SNP were associated with PMN in Europeans, among which *PLA2R1* SNP rs4664308 and *HLA-DQA1* SNP rs2187668 were the most significant [6]. *HLA-DQA1* and PLA2R1 genotypes were also regarded as risk factors in a Spanish study [11]. Saeed pointed out that common mutations in *PLA2R1*, especially rs35771982, were related to PLA2R-positive membranous glomerulopathy in Caucasians caused by *HLA-DQA1*, although *PLA2R1* SNP was not associated with membranous glomerulopathy in African Americans [12]. An Indian study confirmed that *PLA2R1* SNP rs3749119, rs3749117, and rs4664308, and *HLA-DQA1* SNP rs2187668 were significantly associated with PMN, and *HLA-DQA1* SNP rs2187668 was associated with anti-PLA2R positivity [13]. A Japanese study showed that the *PLA2R1* SNP rs35771982, rs3749119, rs2715928, rs16844715, HLA-DRB1*15:01, HLA-DQB1*06:02 were associated with PMN [14]. A Korean study indicated that the *PLA2R1* SNP loci rs35771982 and rs3828323 are associated with the risk of IMN [15]. Cui et al. introduced *PLA2R1* SNP rs4664398 and *HLA-DQA1* SNP rs2187668, which are associated with PMN in the Hebei, Sichuan, and Xinjiang provinces, and found different frequencies of SNP alleles in regional populations and races within China [16]. Another Chinese study involving two cohorts from southern and northwestern China also confirmed that *PLA2R1* SNP rs35771982, rs3749117, and rs4664308 were associated with PMN, and regional otherness was also mentioned [17]. A Study in Harbin demonstrated that the G allele of SNP rs35771982 was a risk allele for PMN [18]. All the aforementioned studies support the results of our study to varying degrees. Furthermore, a 2021 study in Shanghai implied that *PLA2R1* SNP loci rs35771982, rs4664308, and rs3749117 were related to PMN, whereas *HLA-DQA1* SNP rs2187668 was not. Their results for rs2187668 were different from ours and those of previous studies, perhaps because of the small sample size used for genetic analysis [19]. In summary, PMNs are influenced by SNPs, and previous research has mainly focused on PLA2R1 and HLA-DQA1, with the most important loci being rs4664308, rs35771982, and rs2187668.The A allele of rs4664308 is considered a risk allele for PMN, but the pathogenic alleles of rs35771982 and rs2187668 are different in different studies.These three loci were also discussed in this study.Our research results indicate that the G allele of rs35771982 is a risk allele for PMN, which is consistent with the views of Thiri, Raja Ramachandran, and M Saeed. However, C X Tian and Sejoong Kim believe that the C allele of rs35771982 is a risk allele for PMN.Similarly, like most researchers, our study showed that the A allele of rs2187668 is a risk allele for PMN, while the results of M Saeed et al. indicated that the G allele is a risk allele for PMN.This may be due to the differences in research populations, which is why research in different regions and ethnic groups is crucial.

Meanwhile, the gene risk model analysis of the PLA2R1 SNP rs1870102 and rs17831251 and *HLA-DQA1* SNP rs2187668 also made sense in this study. Rs1870102 was strongly associated with PMN in the additive model (OR 93.1983, *P* 0.0061), which indicated that the more A allele carried, the higher the risk of developing PMN. Rs17831251 showed the strongest association with PMN in the dominant and recessive models (OR 0.4259, *P* 0.0424), suggesting that individuals carrying the TT genotype of rs17831251 have a higher risk of PMN. Rs2187668 was associated with PMN in the implicit model (OR 0.2417, *P* = 0.034), indicating that individuals carrying the AA genotype of rs2187668 have a higher risk of PMN than those carrying the GG or GA genotype.

This study demonstrated the interaction of *HLA-DQA1* and *PLA2R1* genes, in which patients carrying the risk allele may have 52.875 times the risk of PMN of those carrying the homozygous protective allele. Previous studies have explored interactions between *PLA2R1* and *HLA* polymorphisms. In 2011, Stanescu reported that homozygotes with two risk alleles (*HLA-DQA1*rs2187668/*PLA2R1*rs4664308 AA/AA) had a risk approximately 80 times that of heterozygotes [6]. Studies conducted in India and China have shown an interaction between rs2187668 and rs4664308 [13,20]. In 2019, a study in a western Chinese Han population showed that the interaction between rs2715918 GA/AA, rs4665143 GA/AA, and rs2187668 GA/AA increased the risk of PMN by 10.61 times [21]. In addition, a study in a German population suggested an interaction between *PLA2R1* SNP rs17830558 and *HLA-DQA1* SNP rs9272729 [22]. These studies indicate that synergism between *PLA2R1* and *HLA-DQA1* increases the risk of MN, which may play a role in further studies aimed at understanding the pathogenesis of PMN.

The 2021 KDIGO guidelines recommend different immunotherapy regimens for patients with MN with different risk stratifications, and the serum anti-PLA2R-Ab titer was used as a reference criterion for risk stratification. Our previous studies also demonstrated that PLA2R IgG and IgG 4 antibody titers are closely associated with PMN [23–26]. Moreover, there is no precise reference standard for the choice of immunosuppressive treatment regimen in moderate- and high-risk patients in the 2021 KDIGO guidelines. A study in 2018 showed that SNP loci on *HLA-DQA1* were significantly associated with a higher level of PLA2R antibody, and HLA-DRB1*1502 was associated with renal outcomes in patients with PMN [27]. A Spanish study in 2014 first demonstrated the relationship between *PLA2R1* and *HLA-DQA1* polymorphisms and IMN response to immunosuppressive therapy; the *PLA2R1* SNP rs4664308 and *HLA-DQA1* SNP rs2187668 seemed to respond to immunosuppressive therapy; and this tendency became more significant when the two SNP loci were combined [11]. This study conducted a preliminary exploration of the relationship between gene polymorphisms and treatment responsiveness of PMN. We first analyzed the correlation between seven SNPs and proteinuria remission at the 12th month in patients who received immunosuppressive therapy and found that *PLA2R1* SNP rs35771982 was associated with treatment response. To identify which immunosuppressive treatment regimen was associated with proteinuria remission, we grouped the patients according to treatment regimen and analyzed the correlation of SNP with treatment response separately. The RTX group showed four related SNP loci: rs3792189, rs3792192, rs17831251, and 35771982, but no similar correlation was observed in the CTX group. We speculate that this is because the key role of RTX is to inhibit B cell proliferation, which is a link in the body’s immune response pathway. At the same time, the protein encoded by the HLA-DQA1 gene plays an important role in immune presentation, and there may be some intersection between the two.This may provide clues for the choice of an immunosuppressive regimen for PMN in further studies.

In conclusion, we found that polymorphisms of the *PLA2R1* and *HLA-DQA1* genes were significantly associated with PMN and that the gene interaction between *PLA2R1* and *HLA-DQA1* increased the risk of PMN. At the same time, the treatment response of patients was found to be related to *PLA2R1* SNP, especially in the RTX group, which may provide a clue for the choice of treatment for patients with PMN. However, because of the limitations of the single center, single ethnicity, and insufficient follow-up time, further exploration of more SNP loci is required in larger study populations, different areas, and multicenter studies.

## Supporting information

S1 Table Clinical data of baseline and 12-month follow-up in PMN. (SAV)

S2 Table SNPs in PMN patients and control group. (XLSX)

S3 Table Correlation analysis between SNPs and remission in PMN patients. (XLSX)

S1 Checklist STROBE statement. (DOC)

S1 File S1 Table. Representative research results cited in this paper. (DOCX)
