# Supplementary material for: PLA2R1 and HLA-DQA1 SNP in patients with primary membranous nephropathy
Source: PLoS One. 2025 Aug 22;20(8):e0328234. doi: 10.1371/journal.pone.0328234 (PMC12373238; doi:10.1371/journal.pone.0328234)
Supplement: S1 Checklist — (DOC) [file pone.0328234.s004.doc]

STROBE Statement—checklist of items that should be included in reports of observational studies

|  | Item No | Recommendation |
| --- | --- | --- |
| **Title and abstract** | 1 | (*a*) Indicate the study’s design with a commonly used term in the title or the abstract P1 |
|  |  | (*b*) Provide in the abstract an informative and balanced summary of what was done and what was found P2-P3 |
| Introduction | | |
| Background/rationale | 2 | Explain the scientific background and rationale for the investigation being reported P3-P4 |
| Objectives | 3 | State specific objectives, including any prespecified hypotheses P4.line 79-81 |
| Methods | | |
| Study design | 4 | Present key elements of study design early in the paper P4-P6 |
| Setting | 5 | Describe the setting, locations, and relevant dates, including periods of recruitment, exposure, follow-up, and data collection P4-P5.line 84-94 |
| Participants | 6 | (*a*) *Cohort study*—Give the eligibility criteria, and the sources and methods of selection of participants. Describe methods of follow-up P4-P5,line 84-94 |
|  |  | (*b*) *Cohort study*—For matched studies, give matching criteria and number of exposed and unexposed NA |
| Variables | 7 | Clearly define all outcomes, exposures, predictors, potential confounders, and effect modifiers. Give diagnostic criteria, if applicable P4-P6 |
| Data sources/ measurement | 8* | For each variable of interest, give sources of data and details of methods of assessment (measurement). Describe comparability of assessment methods if there is more than one group P5,line 95-102 |
| Bias | 9 | Describe any efforts to address potential sources of bias NA |
| Study size | 10 | Explain how the study size was arrived at NA |
| Quantitative variables | 11 | Explain how quantitative variables were handled in the analyses. If applicable, describe which groupings were chosen and why Line 84-94.1ine 104-108 |
| Statistical methods | 12 | (*a*) Describe all statistical methods, including those used to control for confounding Line 109-116 |
|  |  | (*b*) Describe any methods used to examine subgroups and interactions NA |
|  |  | (*c*) Explain how missing data were addressed NA |
|  |  | (*d*) *Cohort study*—If applicable, explain how loss to follow-up was addressed NA |
|  |  | (*e*) Describe any sensitivity analyses NA |

Continued on next page

| Results | | |
| --- | --- | --- |
| Participants | 13* | (a) Report numbers of individuals at each stage of study—eg numbers potentially eligible, examined for eligibility, confirmed eligible, included in the study, completing follow-up, and analysed Line 124-130 |
|  |  | (b) Give reasons for non-participation at each stage NA |
|  |  | (c) Consider use of a flow diagram NA |
| Descriptive data | 14* | (a) Give characteristics of study participants (eg demographic, clinical, social) and information on exposures and potential confounders Line 124-130 |
|  |  | (b) Indicate number of participants with missing data for each variable of interest NA |
|  |  | (c) *Cohort study*—Summarise follow-up time (eg, average and total amount) NA |
| Outcome data | 15* | *Cohort study*—Report numbers of outcome events or summary measures over time NA |
|  |  | *Case-control study—*Report numbers in each exposure category, or summary measures of exposure Line 124-130,line 197-203 |
|  |  | *Cross-sectional study—*Report numbers of outcome events or summary measures NA |
| Main results | 16 | (*a*) Give unadjusted estimates and, if applicable, confounder-adjusted estimates and their precision (eg, 95% confidence interval). Make clear which confounders were adjusted for and why they were included Line 122-206 |
|  |  | (*b*) Report category boundaries when continuous variables were categorized Line 104-108 |
|  |  | (*c*) If relevant, consider translating estimates of relative risk into absolute risk for a meaningful time period NA |
| Other analyses | 17 | Report other analyses done—eg analyses of subgroups and interactions, and sensitivity analyses NA |
| Discussion | | |
| Key results | 18 | Summarise key results with reference to study objectives Line 301-305 |
| Limitations | 19 | Discuss limitations of the study, taking into account sources of potential bias or imprecision. Discuss both direction and magnitude of any potential bias Line 305-307 |
| Interpretation | 20 | Give a cautious overall interpretation of results considering objectives, limitations, multiplicity of analyses, results from similar studies, and other relevant evidence Line 301-305 |
| Generalisability | 21 | Discuss the generalisability (external validity) of the study results NA |
| Other information | | |
| Funding | 22 | Give the source of funding and the role of the funders for the present study and, if applicable, for the original study on which the present article is based NA |

*Give information separately for cases and controls in case-control studies and, if applicable, for exposed and unexposed groups in cohort and cross-sectional studies.

**Note:** An Explanation and Elaboration article discusses each checklist item and gives methodological background and published examples of transparent reporting. The STROBE checklist is best used in conjunction with this article (freely available on the Web sites of PLoS Medicine at http://www.plosmedicine.org/, Annals of Internal Medicine at http://www.annals.org/, and Epidemiology at http://www.epidem.com/). Information on the STROBE Initiative is available at www.strobe-statement.org.
