# Supplementary material for: PLA2R1 and HLA-DQA1 SNP in patients with primary membranous nephropathy
Source: PLoS One. 2025 Aug 22;20(8):e0328234. doi: 10.1371/journal.pone.0328234 (PMC12373238; doi:10.1371/journal.pone.0328234)
Supplement: S1 File — (DOCX) [file pone.0328234.s005.docx]

**Supporting information**

**S1 Table. Representative research results cited in this paper.**

| **S1 Table. Representative research results cited in this paper.** | | | | | |
| --- | --- | --- | --- | --- | --- |
| Stanescu | 2011 | French, Dutch, and British | Risk HLA-DQA1 and PLA(2)R1 alleles in idiopathic membranous nephropathy | PLA2R,HLA-DQA1 | rs4664308 ,rs2187668 |
| [Yu-Huei Liu](https://pubmed.ncbi.nlm.nih.gov/?term=Liu+YH&cauthor_id=20937089) | 2010 | Taiwan,China | Association of phospholipase A2 receptor 1 polymorphisms with idiopathic membranous nephropathy in Chinese patients in Taiwan | PLA2R | rs35771982 |
| [Jingyuan Xie](https://pubmed.ncbi.nlm.nih.gov/?term=Xie+J&cauthor_id=32231244) | 2020 | East Asia, Europe | The genetic architecture of membranous nephropathy and its potential to improve non-invasive diagnosis | NFKB1,IRF4,PLA2R1,DQA1,DRB1 | rs230540,rs9405192, rs17831251,DRB1*1501(East Asia),DQA1*0501(Europe),DRB1*0301(both) |
| Gemma Bullich | 2014 | Spanish | HLA-DQA1 and PLA2R1 polymorphisms and risk of idiopathic membranous nephropathy | PLA2R,HLA-DQA1 | rs4664308 A,rs2187668 |
| M Saeed | 2014 | Europe | PLA2R-associated membranous glomerulopathy is modulated by common variants in PLA2R1 and HLA-DQA1 genes | PLA2R,HLA-DQA1 | rs35771982 ,rs2187668 |
| Raja Ramachandran | 2016 | South Asia | PLA2R antibodies, glomerular PLA2R deposits and variations in PLA2R1 and HLA-DQA1 genes in primary membranous nephropathy in South Asians | PLA2R,HLA-DQA1 | rs3749119,rs35771982,rs3828323,rs4664308,rs2187668 |
| Myo Thiri | 2016 | Japan | High-density Association Mapping and Interaction Analysis of PLA2R1 and HLA Regions with Idiopathic Membranous Nephropathy in Japanese | PLA2R,DRB1,DQB1 | rs3749119 C,rs35771982 G,DRB1*1501,DQB1*0602 |
| Sejoong Kim | 2010 | Korea | Single nucleotide polymorphisms in the phospholipase A2 receptor gene are associated with genetic susceptibility to idiopathic membranous nephropathy | PLA2R | rs35771982 ,rs3828323 |
| C X Tian | 2020 | China | Association of SNPs in PLA2R1 with idiopathic and secondary membranous nephropathy in two Chinese cohorts | PLA2R | rs35771982,rs3749117,rs4664308 |
| Fan Wang | 2021 | China | PLA2R1 and HLA-DQA1 gene variations in idiopathic membranous nephropathy in South China | PLA2R,HLA-DQA1 | rs35771982,rs4664308,rs3749117,rs2187668 |
| Jicheng Lv | 2013 | China | Interaction between PLA2R1 and HLA-DQA1 variants associates with anti-PLA2R antibodies and membranous nephropathy | PLA2R,HLA-DQA1 | rs4664308,rs2187668 |
| Wei Wang | 2019 | China | Interaction between PLA2R1 and HLA-DQA1 variants contributes to the increased genetic susceptibility to membranous nephropathy in Western China | PLA2R,HLA-DQA1 | rs2715918,rs4665143,rs2187668 |
| Stanescu | 2011 | French, Dutch, and British | Risk HLA-DQA1 and PLA(2)R1 alleles in idiopathic membranous nephropathy | PLA2R,HLA-DQA1 | rs4664308 ,rs2187668 |
